# Multi-layer relationships between psychological symptoms and life adaptation among humidifier disinfectant survivors

**DOI:** 10.3389/fpsyg.2022.890122

**Published:** 2022-09-12

**Authors:** Min Joo Lee, Hun-Ju Lee, Hyeyun Ko, Seung-Hun Ryu, Sang Min Lee

**Affiliations:** ^1^Department of Education, Korea University, Seoul, South Korea; ^2^University Industry Foundation, Yonsei University, Seoul, South Korea; ^3^Humidifier Disinfectant Health Center, National Institute of Environmental Research, Seogu, South Korea

**Keywords:** humidifier disinfectant survivors, psychological symptoms, adaptive function, canonical correlation analysis, Achenbach system of empirically based assessment

## Abstract

In April 2011, the Korea Centers for Disease Control and Prevention (CDC) announced the results of an epidemiological investigation that an unknown cause of lung disease that occurred throughout Korea was caused by humidifier disinfectants. The unprecedented social catastrophe caused by humidifier disinfectants, a household chemical, has so far reported 1,784 deaths and 5,984 survivors in South Korea. This study was designed to investigate the multi-layer relationships between psychological symptoms and adaptive functioning in survivors of the Humidifier disinfectants in South Korea caused by chemical toxic substances. Specifically, this study aimed to explore how psychological symptoms affect actual interpersonal relationships and job adjustment with two variable sets, six internalizing and externalizing subscales, and three adaptation subscales. A total of 224 survivors recruited from a program to support humidifier disinfectant survivors by the government participated in this study. This research was approved by the Institutional Review Board of one of the Universities in South Korea. The age range of the participants was 18–73 years (*M* = 42.23, *SD* = 10.90), 37.1% (*n* = 83) were male, and 62.9% (*n* = 141) were female. The participants responded to the Adult Self-Report (ASR) of the Achenbach System of Empirically Based Assessment (ASEBA). A Canonical Correlation Analysis (CCA) generated three unique patterns in the relationships between psychological symptoms and adaptive functions. Humidifier disinfectant survivors in the first pattern were more vulnerable to psychological symptoms and showed maladaptive functioning in life. Survivors in the second pattern showed intrusive behaviors and appeared to be adaptive in relationships with friends. Finally, survivors in the third pattern showed aggressive behaviors and reported poor partner relationships while showing good relationships with friends. The practical implications of the interventions are also discussed.

## Introduction

Chemicals have become necessities of modern society and are sold in many places in our daily lives. These products are not only useful to humans but also harm the human body at the same time. The humidifier disinfectant sold between 1994 and 2011 in Korea was developed to prevent bacterial diseases caused by microorganisms; however, it caused fatal lung damage to random consumers who purchased it (Choi et al., 2018; Ryu et al., 2019). Humidifier disinfectants include polyhexamethylene guanidine phosphate (PHMG-P), polyhexamethylene guanidine hydrochloride (PHMG-H), oligo (2-(2-ethoxy)ethoxyethyl) guanidinium (PGH), chloromethylisothiazolinone (CMIT), methylisothiazolinone (MIT), etc., and products including CMIT and MIT had been sold since 1994 (Yoon et al., 2017; Ryu et al., 2019).

In 2011, the Korea Center for Disease Control and Prevention (KCDC) and the Korean Ministry of Environment reported that inhalation of a humidifier disinfectant was associated with lung damage such as lung fibrosis symptoms in animal experiments (Park et al., 2017). The number of victims of humidifier disinfectant use increased from 2008 to 2011 and showed a marked increase between 2010 and 2011. This was the first reported unprecedented biocide death from household chemicals in South Korea, and the impact and extent of the damage are not yet clear (Choi et al., 2018). As of November 2021, the number of people who apply for damage was 7,642 including 1,740 deaths (Comprehensive Humidifier Disinfectant Damage Support Portal, 2021).

Humidifier disinfectant damage adversely affects not only individuals’ physical health but also their psychological health. Among the victims, 57.5% experienced depression, 55.1% experienced guilt and self-blame, 54.3% were anxious, 27.6% had suicidal thoughts, and 11% attempted suicide (Leem et al., 2020). Ko et al. (2021) compared the mental health status of 228 victim-survivors with 228 members of the general population from 2018 to 2021. They reported that victim survivors had much higher scores on anxiety, depression, atrophy, physical discomfort, thinking problems, attention problems, and aggressive behavior than the general population. In addition, it was found that victim survivors with high economic status showed much higher psychological distress than their counterparts (e.g., low and middle classes), and it was revealed that economic stability and psychological pain can be independent in the sample of victims of humidifier disinfectants. Thus, it is important to carefully examine the mental health status of humidifier disinfectant survivors by utilizing a more comprehensive psychological assessment.

Most psychological symptom assessments are categorized as the ASEBA (Achenbach System of Empirically Based Assessment), which is used to evaluate the adaptive states and problem behaviors of adults (Kim et al., 2014). The ASEBA include two psychological syndrome constructs: internalizing and externalizing problem behaviors. While internalizing problem behaviors hide problems inward and internalize and overcontrol problems, externalizing problem behaviors consist of apparent behavioral problems and externalized and under-controlled problems (Achenbach and Rescorla, 2003). There are three subscales for both internalizing and externalizing. The internalizing subscales are anxious/depressed, withdrawn, and somatic. On the other hand, the externalizing subscales include aggressive behavior, rule-breaking behavior, and intrusiveness. Internalizing and externalizing subscales can differentially contribute to the overall problem behavior of adults.

In addition to psychological symptoms, the ASEBA also includes the Life Adaptation Scale, which consists of relationships with friends, spouses/partners, as well as job adjustment. Therefore, it is possible to closely analyze how psychological symptoms may affect actual functioning in daily life. In Italy, the ASR was conducted to monitor changes in the first 4 weeks of isolation in order to assess the mental health status of young people during the COVID-19 pandemic (Parola et al., 2020). The results showed a significant increase in both internalizing and externalizing behavior and a decrease in social relationships due to isolation and containment. Jang et al. (2020) also utilized the ASR to examine the psychological symptoms of earthquake victims in Nepal and reported that more than half of the adult victims of the Nepal earthquake showed symptoms of post-traumatic stress disorder (PTSD). In addition, more than half of them had internalization problems requiring clinical intervention, and a significant number had externalization problems. The research also reported that parents’ internalizing and externalizing symptoms are significantly correlated with children’s PTSD symptoms; in other words, the occurrence of a disaster is not only related to one’s psychological pain but also to the psychological pain of close relationships such as family. For example, victims of the Fukushima incident experienced psychological pain as well as the dissolution of their families or complained of extreme stress in parenting in structurally difficult conditions (Tsujiuchi, 2021). Therefore, social disasters have a psychologically and physically impact upon relationships, such as those within families or within interactions with others.

This study aimed to explore the multi-layer relationships between psychological symptoms (both internalizing and externalizing) and maladaptation factors in daily life (e.g., friends, family, and work adjustment). Specifically, this study aimed to explore how psychological symptoms affect actual interpersonal relationships and job adjustment by utilizing a canonical correlation analysis between two variable sets, six internalizing and externalizing subscales, and three adaptation subscales. A canonical correlation analysis (CCA) predicts the simultaneous correlation between two or more independent variables and two or more dependent variables. As the multivariate statistical analysis, a CCA was used for the observation and quantification of associations between two sets of measurements (Härdle and Simar, 2015). Thus, the results of this study can systematically reveal how individuals’ six externalization and internalization psychological symptoms function with actual life adjustments in humidifier disinfectant survivors. By examining the patterns of how survivors’ mental health problems manifest in real life, we can identify important variables when designing individualized intervention strategies for survivors of humidifier disinfectants. The research question is as follows. How does psychological symptom variable sets associate to life adaptation sets among humidifier disinfectant survivors?

## Materials and methods

### Participants

In March 2021, this research was approved by the Institutional Review Board of one of the Universities in South Korea. The participants were recruited from a program to support humidifier disinfectant survivors by the government in South Korea. Humidifier disinfectant survivors voluntarily completed the mental health questionnaire through the portal website for supporting survivors. The study used data from 224 survey respondents who finished their responses, out of the total participants. The age range of the participants was 18–73 years (*M* = 42.23, *SD* = 10.90), 37.1% (*n* = 83) were male, and 62.9% (*n* = 141) were female. Using the G*Power 3.1 program, the number of samples in this study was found to be 111 when the significance level required for CCA analysis, the medium effect size was 0.3 and the variable was set to a total of 9, indicating that the minimum number of samples in this study was satisfied.

### Measures

The Adult Self-Report (ASR) of the Achenbach System of Empirically Based Assessment (ASEBA) was adopted to comprehensively assess the adaptive and maladaptive functioning of survivors. The ASR consists of 120 items with three rating levels: *0 = not true*, *1 = somewhat or sometimes true*, *and 2 = very true or often true* about experiences of the preceding 6 months. The ASR can be divided into two categories: internalizing and externalizing problem behaviors. While internalizing problem behaviors include anxiety/depression (18 items), withdrawal (nine items), and somatic complaints (12 items), externalizing problem behaviors include aggressive behavior (15 items), rule-breaking behavior (14 items), and intrusive behavior (six items). A sample item from the anxious/depressed subscale is “I feel lonely,” and another is “I cry a lot.” These questions are related to feeling emotionally depressed and overly worried or anxious. “I would rather be alone than with people” and “I am not liked by others” are two sample items from the withdrawn sub-scale, which includes questions about withdrawal and inactive attitudes as well as not exhibiting interest in the environment. The somatic complaints subscale measures complaining of various physical symptoms even though there is no clear medical cause, “I feel dizzy or lightheaded” and “I feel tired without good reason” are the sample items. This aggressive behavior measures verbal and physical destructive and aggressive behavior or hostile attitudes, “I damage or destroy my things” and “I break rules at work or elsewhere” are the sample items. The rule-breaking behavior subscale evaluates behavior that impulsively acts on problematic behaviors that do not comply well with rules or go against social norms in the workplace or society, and “I damage or destroy my things” and “I break rules at work or elsewhere” are the sample items. The intrusive subscale accesses behavior that annoys or interferes with others; examples include “I brag” and “I attempt to attract a lot of attention.”

The adaptive functioning subscales of the ASR consist of the relationships with friends (four items), spouse/partner (eight items), and job adjustment (eight items). The adaptive functioning scale consists of questions to determine the subject’s level of adaptation in each living environment. The adaptive functioning scale assesses the degree of overall adaptability in terms of the subject’s capacity to build connections and carry out duties at home, school, and work.

### Statistical analysis

A Canonical Correlation Analysis (CCA) was conducted to explore the relationships between six problem behaviors and three adaptive functioning variables. As suggested by Baggaley (1981) and Thompson (1991), the CCA is a multivariate statistical method that can analyze two variable sets, with each set consisting of two or more variables. In this study, six problem behaviors were used as multivariate independent variable profiles and three adaptive functions (Friend, Spouse/Partner, and Job Adjustment) were used as multivariate dependent variable profiles.

## Results

When analyzing the data, researchers strived to maintain neutral and objective perspective by excluding their own bias. In order to maintain objectivity, various previous studies were reviewed to explore and broaden the understanding of the participants’ context. Descriptive statistics for the psychological syndrome and adaptive functioning subscales are listed in Table 1. The CCA tested the differential predictive validity of the psychological symptoms for adaptive functioning. The analysis of the dependent variables of the three adaptive functions and the independent variables of the six psychological syndromes demonstrated three canonical functions (see Table 2). All three canonical correlations between the two sets of variables were statistically significant, with canonical coefficients (*Coeff*) and squared canonical correlation (*R_c_^2^*) effect sizes of 68.6, 21.8, and 10.8%, respectively. Because Wilks’ λ represents the amount of variance not explained by variable sets, by taking 1 − *λ*, the full model effect size was yielded in an *R_c_^2^* metric. In addition, three functions had interpretable squared canonical correlation effect sizes of 59.8, 12.3, and 10.8%, respectively. Thus, all three functions in the canonical model should be interpreted to explain a reasonable number of variable sets.

**Table 1:** Inter-correlation matrix with descriptive statistics for research variables.

Variables	1	2	3	4	5	6	7	8	9
1. Anxious/Depressed	1								
2. Withdrawn	0.645^**^	1							
3. Somatic complaints	0.712^**^	0.591^**^	1						
4. Aggressive behavior	0.648^**^	0.587^**^	0.558^**^	1					
5. Rule-breaking behavior	0.527^**^	0.548^**^	0.479^**^	0.616^**^	1				
6. Intrusive	0.329^**^	0.198^**^	0.250^**^	0.518^**^	0.449^**^	1			
7. Friend	−0.206^**^	−0.402^**^	−0.223^**^	−0.200^**^	−0.149^*^	−0.014	1		
8. Spouse/Partner	−0.364^**^	−0.359^**^	−0.316^**^	−0.396^**^	−0.307^**^	−0.106	−0.306^**^	0.1	
9. Job adjustment	−0.416^**^	−0.411^**^	−0.409^**^	−0.359^**^	−0.363^**^	−0.209^**^	−0.214^**^	0.413^**^	1
*M*	0.856	0.747	0.676	0.628	0.219	0.307	2.459	1.141	1.376
*SD*	0.431	0.422	0.489	0.373	0.243	0.325	0.664	0.346	0.255

* *p* < 0.05 and

** *p* < 0.001.

**Table 2:** Canonical solution for adaptive functioning predicting psychological symptoms.

	Function 1			Function 2			Function 3			
Variables	Coef	*r_s_*	*r_s_^2^ (%)*	Coef	*r_s_*	*r_s_^2^ (%)*	Coef	*r_s_*	*r_s_^2^ (%)*	*h^2^ (%)*
**Independent variables**										
1. Anxious/Depressed	0.361	0.878	77.2	0.745	0.238	5.7	0.103	0.016	0.0	82.9
2. Withdrawn	0.315	0.854	73.0	−0.869	−0.375	14.1	−0.963	−0.325	10.5	97.6
3. Somatic complaints	0.141	0.778	60.6	0.295	0.173	3.0	−0.421	−0.177	3.1	66.7
4. Aggressive behavior	0.119	0.766	58.7	−0.853	−0.186	3.9	1.199	0.478	22.9	85.5
5. Rule-breaking behavior	0.308	0.777	60.4	0.441	0.197	3.9	0.356	0.241	5.8	70.1
6. Intrusive	−0.076	0.340	11.5	0.499	0.402	16.1	−0.397	0.121	1.5	29.1
*R*_c_			75.4			33.2			29.0	
*R*_c_^2^			56.9			11.0			8.4	
**Dependent variables**										
7. Friend	−0.142	−0.402	16.2	0.762	0.729	53.2	0.670	0.554	30.7	99.9
8. Spouse/Partner	−0.445	−0.759	57.6	0.453	0.359	12.9	−0.919	−0.544	29.6	100.0
9. Job adjustment	−0.680	−0.890	79.3	−0.730	−0.387	14.9	0.467	0.241	5.8	100.0

Coef, standardized canonical function coefficient; *r*_s_, structure coefficient or canonical loading; *r*_s_^2^, structure coefficient squared or variance explained; *h*^2^, communality coefficient; *R*_c_, canonical correlation coefficient; and *R*_c_^2^, squared canonical correlation coefficient. Underline for highlight numbers greater than absolute value of 0.4.

The full model across all functions was statistically significant, with Wilks*’ λ* = 0.314, *F*(21, 626.53) = 14.815, *p* < 0.001. Wilks’ *λ* represents the inverse effect size or the amount of variance unexplained by the model. Therefore, taking 1 – *λ*, 1–0.314 = 0.686 = *R_c_^2^*, the overall effect for the full model was obtained. These results indicate that the full model was statistically significant and had a large effect size that explained 68.6% of the variance shared between the variable sets. The hierarchical arrangement of functions for statistical significance was deduced using dimension reduction analysis in the CCA. As previously mentioned, the full model was statistically significant with Wilks’ *λ* = 0.314, *F*(21, 626.53) = 14.815, *p* < 0.001. Functions 2–3 showed statistical significance, with Wilks’s *λ* = 0.782, *F*(12, 438.00) = 4.770, *p* < 0.001. The only test for Function 3 was also statistically significant, Wilks’s *λ* = 0.892, *F*(5, 222.00) = 5.321, *p* < 0.001.

Table 2 shows the standardized canonical function, structure, squared structure, and communality coefficients for all variables across all functions. The communality coefficients in the last column are the sum of the variables’ squared structural coefficients (*r_s_*). The communality coefficients can be interpreted as a contributing indicator of the variables across functions. Often times, communality (*h^2^*) above 45% is regarded as the highest level of usefulness in the model. All communalities of independent and dependent variables in the present study proved to be highly useful in explaining the models, with values well over 45%.

Absolute values of the structure coefficient (*r_s_*) greater than 0.40 were underlined for emphasis (Sherry and Henson, 2005). The structure coefficient of Function 1 indicated that the five psychological symptoms were important contributors to the synthetic dependent variables. The results for the independent variables of Function 1 showed that five variables, that is Anxious/Depressed (+), Withdrawn (+), Somatic Complaints (+), Aggressive behavior (+), and Rule-Breaking Behavior (+) subscales were useful contributors to the synthetic dependent variables: Friend (−), Spouse/Partner (−), and Job Adjustment (−) subscales. Furthermore, all structure coefficients of the synthetic independent variable set had a positive value and were negatively related to all adaptive functioning variables. This result was consistent with the theoretical hypothesis, in which psychological syndromes are negatively related to adaptive functioning in social relationships (Walton and Wilson, 2018) and in work place (Ford et al., 2011).

**Figure 1:**
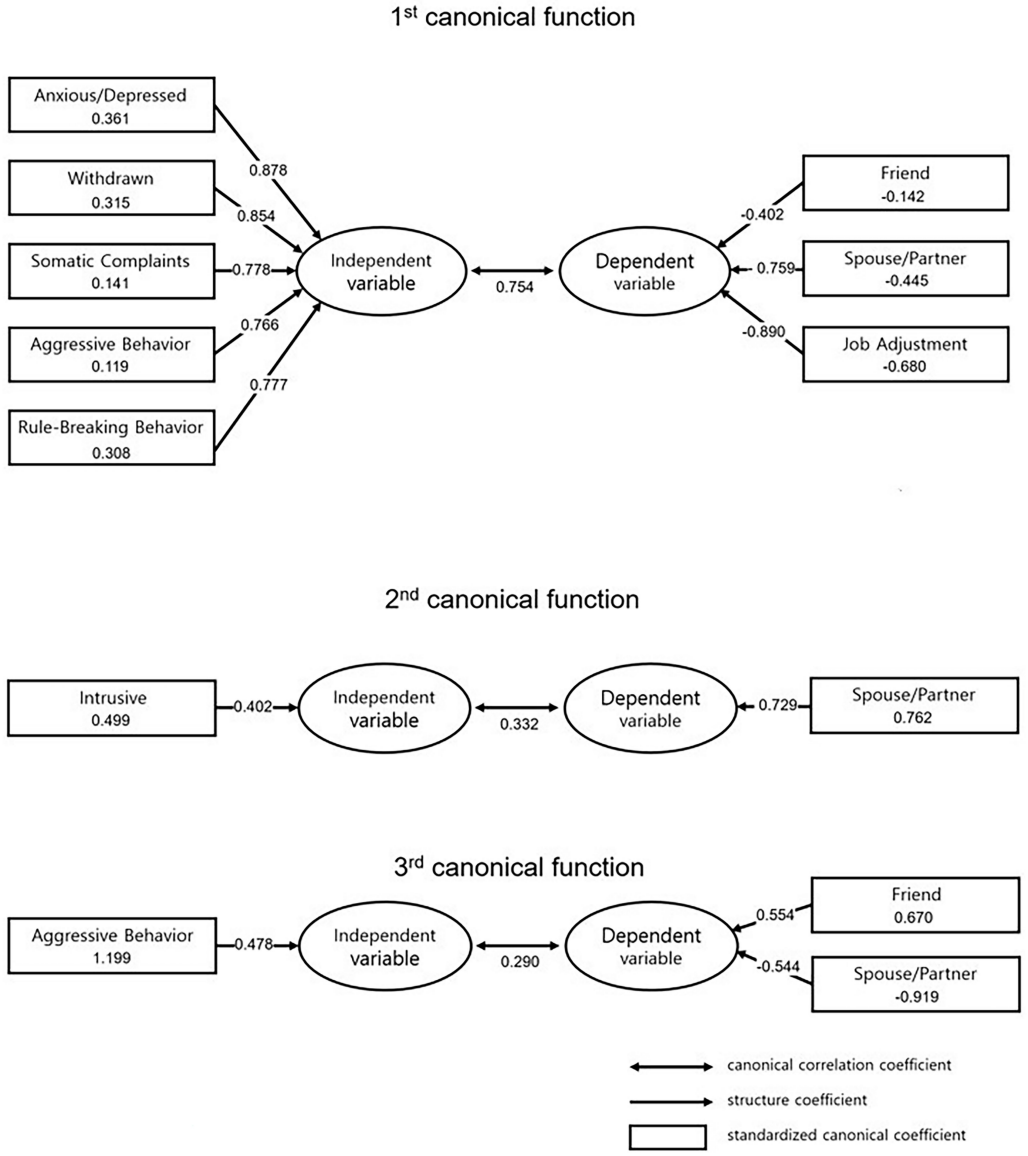
Illustration of the first, second, and third function in a Canonical Correlation Analysis (CCA).

In Function 2, the only significant independent variable was the Intrusive subscale (+) and the only significant dependent variable was the Friend (+) subscale. The Intrusive subscale was positively related to the Friend subscale, which meant that higher intrusive behavior scores indicated better functioning in the Friend relationship. A large body of research has reported an association of Intrusive behavior with friendship (Rubin et al., 2006; Cook and Fletcher, 2012). These findings mainly focused on intrusive behaviors as a factor in externalizing problems and malfunctions of adaptive variables. On the other hand, the results from Function 2 in the present study showed that intrusive behavior corresponding to withdrawal could have a positive effect on friendship. This result could be supported by research on the adverse effects of shyness/withdrawal on friendships in various aspects (Rubin et al., 2006). The result of Function 3 suggested that aggressive behavior (+) was negatively related to the relationship with the spouse or partner (−) but positively related to relationships with friends (+). Thus, this finding revealed that aggressive behavior worsens relationships with spouses/partners. On the other hand, aggressive behavior was positively related to relationships with friends (see Figure 1).

## Discussion

The research question of the present study is as follows: how does psychological symptom variable sets associate to life adaptation sets among humidifier disinfectant survivors? To answer the research question, the present study examined the relationship between psychological symptom subscales and life adaptation subscales using a CCA. The psychological symptom subscales were considered as independent variables, and life adaptation subscales were considered as dependent variables in the CCA, in which the relationship between two variable sets was modeled. According to the results of the canonical correlation, three unique patterns of relationships between psychological symptoms and life adaptation were found. The main statistic of the CCA is aimed at maximizing the correlation between the two synthetic variables (Sherry and Henson, 2005). The CCA derives as many canonical functions as there are sets of variables in the smaller set. The Function 1 was created to maximize the canonical correlation between the five independent variables (Anxious/Depressed, Withdrawn, Somatic Complaints, Aggressive Behavior, and Rule-Breaking Behavior) and the dependent variables (Friend, Spouse/Partner, and Job Adjustment). After excluding the variance of Function 1, Function 2 was created to maximize the other canonical correlations between the two other variable sets. In other words, the second function was created under the condition that these new synthetic variables were perfectly uncorrelated with all the other preceding functions. Function 2 concerned Intrusive Behavior and Friends. Finally, Function 3 was created to correlate two more variable sets as strongly as possible with the remaining variance in the observed variables. Function 3 deduced the correlation between Aggressive Behavior in the independent variable set, and Spouse/Partner and Friend in the dependent variable set.

Function 1 shows individuals who have both internalizing (i.e., high scores on Anxious/Depressed, Withdrawn, and Somatic Complaints) and externalizing symptoms (i.e., Aggressive Behavior and Rule-Breaking Behavior). These individuals presented maladaptive functioning in life adjustment (i.e., low scores on relationships with Friends and Partners and Job Adjustment). These results show how painfully humidifier disinfectant survivors suffer from a myriad psychological problems (Ko et al., 2021). These results are consistent with those of previous studies (e.g., Dell’Osso et al., 2012; Lee et al., 2017; Tian et al., 2020) that reported a significant relationship between psychological symptoms (e.g., posttraumatic stress symptoms, depression, and anxiety) and maladaptive behaviors (e.g., problematic Internet use, alcohol or drug abuse, no self-scare, risk-taking behavior, and negative coping) among the sample of disaster survivors. Since disasters are mostly unpredictable, survivors are in a state of shock, have a tendency to deny the loss, and try to escape from reality. In a denial state, survivors are vulnerable to stress, anxiety, and maladaptive reactions (Makwana, 2019). As humidifier disinfectant disasters occur in the family dimension (Park and Kwon, 2020), humidifier disinfectant survivors could be more vulnerable to psychological symptoms and show maladaptive functioning. As each family member witnesses and experiences the loss caused by humidifier disinfectants, the atmosphere in the family becomes depressed, anxious, and lethargic, leading to the disruption of family bonds and feelings of insecurity at home (Peek, 2008; Figley and Kiser, 2013). In addition, a lack of social resources aggravates survivors’ mental status and life adaptation (Norris et al., 2002a). Therefore, there needs to be active intervention at the societal and community levels to target the recovery of mental health as well as the life adaptation of humidifier disinfectant survivors (Norris et al., 2002b).

As previously mentioned, Function 2 was created using the remaining variance after excluding Function 1. Function 2 presents individuals who exhibit intrusive behaviors (high scores on the Intrusive scale). These individuals appear to be adaptive in their relationships with friends. Considering the sample items of intrusive behaviors (e.g., bragging, demanding attention, showing off, talking too much, and being loud), individuals with high scores in intrusive behaviors can be seen as showing an overreaction to making friends. Yet, these individuals could be seen as sociable to others. Nonetheless, when individuals brag and boast consistently, their relationship with friends eventually deteriorates (Yager, 2010). Therefore, there is a need for education or counseling on how to appropriately establish and maintain good connections with friends.

Function 3 is created with the remaining variance after excluding Functions 1 and 2. Function 3 describes individuals who exhibit aggressive behaviors (high scores on the Aggressive Behavior scale). These individuals reported poor partner relationships while showing good relationships with friends. These results could be explained by resource control theory (RCT; Hawley, 1999). According to RCT, individuals who present high levels of aggressiveness can achieve better social outcomes than non-aggressive individuals when they balance high levels of coercive strategies (e.g., aggression) with high levels of prosocial strategies (e.g., cooperation; Hawley et al., 2007, 2009). People who employ both coercive and prosocial strategies are likely to use relationships instrumentally to attain their goals (Hawley et al., 2009). Therefore, it seems that some humidifier disinfectant survivors use both coercive and prosocial strategies to attain their goals. To illustrate, they utilize aggressive behaviors while endeavoring to interact with friends to accomplish a sense of belongingness that cannot be satisfied in partner relationships.

Even though they showed good relationships with friends, cautious attention must be paid to the fact that they showed poor partner relationships. Poor partner relationship could be a significant risk factor for chronic depression and suicidal thought and behavior (Patel et al., 2002; Kazan et al., 2016). Moreover, when humidifier disinfectant survivors lose the power to use prosocial strategies, only aggression remains, which might ultimately harm the relationship with friends. Couple or marriage counseling, which aims to lessen aggressive behavior toward each other, is often needed for this group.

There are several limitations of the present study that need to be acknowledged. First, since the participants were all adults, it was difficult to generalize the results to different ages of humidifier disinfectant disaster survivors. It is possible that children and adolescents may show different patterns. Therefore, future studies should expand the age range of the samples to generalize the results. Second, CCA, which is based on Pearson’s *r* correlation statistics (Thompson, 1991), limits the understanding of the causal relationship between psychological symptoms and life adaptation. Further studies should adopt a longitudinal method to establish a true cause-and-effect relationship between psychological symptoms and life adaptation. Knowing the causal relationship will allow for more effective interventions for humidifier disinfectant disaster survivors because it gives hints about what should be intervened first. Third, a self-reported assessment restricts the understanding of each function in a more comprehensive way. For a more comprehensive understanding, future studies need to analyze more objective data, such as the amount of damage compensation, degree of damage to the family member, and counseling records of people in each function.

Despite these limitations, the results of the present study have both clinical and research implications. First of all, the present study provide a clear picture of what to target when intervening in humidifier disinfectant disaster survivors, based on each psychological symptom. For groups that display both internalizing and externalizing problems, the government and society should actively target the recovery of mental health as well as life adaptation. In addition, when designing the supporting system which aims at mental health and life adaptation, delicate and integrative approach is needed to protect humidifier disinfectant disaster survivors from individual and social risk (Jin, 2019). Education or counseling aimed at acquiring skills to form intimate relationships with friends is needed for those showing intrusiveness. Moreover, there should also be a monitoring process to check whether they are maintaining the intimate relationship or not. Couple or marriage counseling, which aims to lessen aggressive behavior toward partners, is needed for those who show such behaviors. Along with lessening the aggressive behavior, their intention, purpose, and emotion behind the aggressive behavior should also be explored. The present study also has research implication. It establishes the foundation of humidifier disinfectant disaster research by providing an empirical evidence for the practical direction of medium-and long term systematic support for the survivors.

## Data availability statement

The raw data supporting the conclusions of this article will be made available by the authors, without undue reservation.

## Ethics statement

The studies involving human participants were reviewed and approved by this research was approved by the Institutional Review Board of Yonsei University (No. 7001988-202104-HR-1178-02l). The patients/participants provided their written informed consent to participate in this study.

## Author contributions

SL: conceptualization, supervision, and project administration. ML: methodology and data curation. HL: formal analysis. HK: investigation. S-HR: raw data management and funding acquisition. ML, H-JL, and HK: writing—original draft preparation. SL, ML, H-JL, and HK: writing—review and editing. All authors contributed to the article and approved the submitted version.

## Funding

This research was supported by the National Institute of Environmental Research, Ministry of Environment (ME) of Republic of Korea (NIER-2021-04-03-001).

## Conflict of interest

The authors declare that the research was conducted in the absence of any commercial or financial relationships that could be construed as a potential conflict of interest.

## Publisher’s note

All claims expressed in this article are solely those of the authors and do not necessarily represent those of their affiliated organizations, or those of the publisher, the editors and the reviewers. Any product that may be evaluated in this article, or claim that may be made by its manufacturer, is not guaranteed or endorsed by the publisher.
